
JOURNAL INFORMATION
==============================
NLM Title Abbreviation: Med Chir Rev
NLM Journal ID: 0034617

The Medico-Chirurgical Review

Publisher: Biomedical Journal Digitization Project

ARTICLE INFORMATION
==============================
PMCID: PMC4966175
PMID: 29917598
Article version: 1
Subjects: Articles

Varieties

Print publication date: 1828 Jul 1
Volume: 9
Issue: 17
First page: 193
Last page: 216
1828 May 10
License: This work is free of known copyright restrictions. For more information, please see PMC Back Issue Digitization.
License URL: https://creativecommons.org/publicdomain/mark/1.0/

==============================

43. OUTLINE OF THE NATURE AND CURE OF STAMMERING, OR HESITATION OF SPEECH. By HENRY M'CORMACK, M.D.

Being in New York, in the year 1826, I learnt that a Mrs. Leigh of that city, had acquired great reputation for the cure of hesitation of speech, such as stammering, &c. I soon found also that Mrs. Leigh had obtained, from several eminent medical men, who were, of course, incapable of lending themselves to a falsehood or deception, certificates of their belief in the reality of Mrs. Leigh's cures. These gentlemen had been admitted into this lady's confidence, and permitted to witness the process of cure, on pledging their solemn promise never to divulge the secret. They therefore gave their attestations to the fact that, the same means invariably produced the same favourable results. As I could glean no satisfactory information on this subject from books, I very zealously applied myself to an investigation of this distressing DEFECT—which investigation occupied my mind, day and night, during a tour through the United States. With equal confidence and satisfaction, I can now assert that the cause of stammering, hitherto so inscrutable, is not only easily understood, but capable of being removed with the utmost facility, by any one acquainted with the said cause, and who will take the trouble to put the simple process of cure into operation. A few words will suffice to convey to the reader the result of my investigation ; a fuller exposition of which will be offered to the public in a small work, now preparing for the press.

During the act of speaking, air must either be passing *out*, or passing *into* the lungs—or, at all events, out or into the mouth—that is, during expiration or inspiration. Most people (who have a perfect command over the organs of speech) can articulate imperfectly and with difficulty during INSPIRATION—but not so the stammerer. In attempting to speak, while *drawing in* the air, or while the lungs are empty, or nearly so, HE WILL not be able to articulate at all ; and not knowing the cause of this inability, he will make repeated, and often convulsive efforts, accompanied with more or less of those hideous distortions of countenance so characteristic of the stammerer, until, by accident, rather than by design, he draws in a full breath, and effects the utterance of his words, while the air is flowing naturally *from* the lungs. I repeat it then, that—STAMMERING ARISES FROM AN ATTEMPT TO SPEAK WHEN THE LUNGS ARE EMPTY, OR WHEN THE STAMMERER IS DRAWING IN HIS BREATH. This habit having been once contracted, generally becomes aggravated by time ; and I need not say that thousands of individuals are almost cut off from social intercourse, and rendered miserable by the said impediment.

From these premises, which I know to be correct, the method of cure will be easily understood. It consists in making the stammerer (if a child, for an adult can do it himself) take in a deep inspiration, and repeat, with the whole force of the *expiration*, the different letters of the alphabet—numerals—monosyllables, *one by one*. This may be prefaced or not, by several hours practice of slow and deep breathing. As for the repetition of the monosyllabic pronunciation, it must be continued for hours, days, or weeks, according to the condition of the patient, such as his age, capacity, strength of lungs, or inveteracy of the impediment. The stammerer must next proceed to the utterance of polysyllables, *during one expiration* :—then short sentences—and, lastly, long sentences ; thus reversing, in fact, the evil habit, until, at length, a new habit is acquired, and the cure effected. In some cases, this desirable object will be accomplished in a few hours—in others, it will require months. In general, a few days, or at most, weeks, will be sufficient.

Having put this discovery to the test of experiment, and completely verified the theory I had formed in my mind, I need hardly say that I choose the pleasure of communicating a blessing to my fellow creatures rather than the acquisition of personal emolument. It is an additional satisfaction to me to reflect, that this discovery of the cause and cure of stammering, was not a mere lucky thought, but the result of physiological investigation.

It is not my intention, in this short notice, to enter on a consideration of certain forms of hesitation of speech, dependent on organic defects, or which occur only when violent passions of the mind are in operation. Neither need I advert to the absurd remedies which have been proposed for this distressing malady —or rather mal-habit. SLOWNESS OF UTTERANCE is the most universal recommendation in such cases. But this, without reference to the BREATHING, above alluded to, will be found totally inadequate to the object in view. The cutting of the frænum linguæ—the Demosthenic practice of addressing the ocean with pebbles in the mouth—speaking with the teeth closed—with the tongue applied to the anterior part of the palate, &c. need not here be discussed. The use of purgative medicines, where the nerves of speech are sympathetically disordered by the digestive organs, is a more rational and effective remedy than any of the above; but this alone will not avail in habitual stammering.

I have the satisfaction of not merely hoping, but of knowing, that this short and simple exposition will, in the hands of my medical brethren, be the means of communicating an invaluable blessing and enjoyment to thousands of my fellow-creatures—of rescuing the community from the harpy claims of mercenary Charlatans—and removing from the medical profession the opprobrium of being unable to do that which is effected by men and even women, unacquainted with the structure, the physiology, or the diseases of the human frame.

### 44. GLEET, OR CHRONIC GONORRHŒA.

In Professor Smith's Philadelphia Monthly Journal for September last, Dr. M'Knight communicates the following observations on the above-mentioned troublesome complaint, elicited in consequence of reading an extract from the Medico-Chirurgical Review on the same subject.

"My experience in the treatment of the above disease is by no means limited. During a practice of eleven years, six and a half of which were spent in the United States army, I have met with every variety of case. It is to be presumed that every surgeon treats the disease in its first stage as one of an inflammatory character. I shall, therefore, confine my observations to its chronic form and to that stage which strictly deserves the name of gleet.

"After the first four or five days, if the proper antiphlogistic remedies have been employed, the discharge is changed from a green, yellow, or greenish yellow, to a light straw colour, which may with great propriety be termed the second stage of the disease. I prescribe the following mixture: tinct. canthar. vesicatoriæ, 6 drachms, balsam copaib. 2 drachms; mix; dose 10 drops morning and evening, gradually increased until the cure is effected, which is generally in a few days.

"In the third stage of the disease, which may be nosologically termed gleet, the discharge is white, more or less in quantity, unattended with ardor urinæ, and, if allowed to progress, followed by pain in the lumbar vertebræ, with general debility. Here I employ the tinct. cantharid. vesicat. alone, or blend it with the balsam for the purpose of disguising the remedy (the balsam alone is not to be relied upon, I having given it in some cases to the extent of a pint without any effect), commencing with 15 drops of the active remedial agent three times a day, and cautiously increasing the quantity until the discharge ceases and the disease is cured.

"In obstinate cases of long standing that have fallen under my notice, I have found the ol. terebinthinæ a useful auxiliary. Chalybeates with a generous diet are highly proper.

"The above practice was taught me by my respected preceptor, Professor Hosack, in 1814, and having succeeded in *every* case to which my attention was directed, has superseded the necessity of hunting after *new* remedies."

### 45. INFLAMMATION OF THE PLACENTA.

On this subject, M. Brachet has pub[illegible]lished a paper in a recent Number of th[illegible] Journal Général, which we shall her[illegible] notice. M. B. remarks that this inflam[illegible]mation is equally dangerous to the mothe[illegible] and the fœtus. By intercepting or de[illegible]ranging the placental circulation, it ma[illegible] destroy the latter quickly, or lead t[illegible] abortion. Where the phlogosis is nc[illegible]

so extensive or intense as to destroy the life of the embryo, it may greatly injure its health and obstruct development, by diminishing, more or less, the nutritive materials supplied by the mother. This inflammation is dangerous to the parent, because it may spread to the uterus, which is a serious consequence. Even in the induction of abortion, it is not devoid of danger to the mother. The causes of this placental inflammation are, blows on the abdomen, falls, shocks, violent contortions of the body, frights, strong mental emotions, &c. Inflammatory affections of the mother, and especially metritis, may also lead to placental inflammation. If, after the application of one or more of these causes, we find a pregnant woman complain of pains in the loins, coming on periodically, but never entirely ceasing, leaving in the intervals a dull and uneasy sensation, we may presume that there is placental inflammation in existence. In such case, abortion is imminent—or, if this do not take place, the life of the fœtus is in danger. We cannot be too early in our endeavours to remedy the evil; and as, in all other inflammations of parenchymatous structures, blood-letting is the most effectual measure. But timid depletion will not succeed. The patient should be kept in the horizontal position, and a large quantity of blood should be detracted. To quietude, position, and venesection, cold drink should be added. But it is not sufficient to remove the more urgent symptoms. Antiphlogistic measures should be continued, till all sense of weight, pain, or uneasiness, is removed from the loins or uterine region, in order that induration of the placenta, the consequence of chronic inflammation, may not succeed the acute form, and thus produce those troublesome adhesions of the placenta to the uterus, which require manual force for separation after delivery.

Dr. Brachet has no doubt that many morbid conditions of the placenta escape our notice, in consequence of the little attention that is paid to the examination of this part after its removal from the uterus.

---

### 46. VOLUMINOUS ABSCESS (SO CALLED) IN THE ABDOMEN.

M. Gaspero, aged 19 years, of weakly constitution, but most dissipated habits, and fond of violent gymnastic exercises, began to complain of debility, loss of appetite, perspirations, and wandering pains about the spine and abdomen, which did not, however, confine him to the house. In the month of September, he became affected with icterus, which soon disappeared; but his health continued to decline. On the *25th October*, he consulted Dr. Cartoni, on account of acute pains in the dorsal portion of the spine, augmented by motion, but not by pressure. He was emaciated—had slow fever and some cough. The physician considered the complaint as inflammation of the spinal marrow, and advised repose, leeches, low diet, &c. On the *2d November*, Dr. C. found the patient complaining of severe pain in the spine and abdomen. The emaciation had increased—the tongue was red—there was much thirst, with slow fever—constipation—burning heat in the interior—cough—quick pulse. It was with some surprise that Dr. Cartoni now discovered a tumour, of considerable size, in the epigastric and umbilical region, evincing a distinct fluctuation throughout its whole extent. The examination led to the belief, that an immense abscess existed. It is unnecessary to say that no remedy proved of any avail, and in a consultation, at which the celebrated Vacca assisted, it was determined to open the abscess, warning the patient and friends of the danger of the operation. On the 19th November, Dr. Cartoni plunged a bistoury into the most depending part of the tumour, and gave exit to a large quantity of pus, mixed with flocculent matters. When about seven pints were drawn off, syncope threatened, and the orifice was closed. At the subsequent dressings, some hydatids came away along with the puriform discharge. The patient died six days after the operation.

*Dissection.* The abdomen contained some quantity of serous effusion. An immense sac, denominated the parietes of an abscess by the authors, was adherent to various viscera, displacing them in different directions, and greatly encroaching on the thoracic organs. This sac contained about twelve pints of fetid fluid, in which swam an immense number of vesicular bodies—in other words, hydatids.—JOURNAL DE PROGRÉS.

*Remarks.* This case, which is by no means uninteresting in itself, is calculated to excite some reflexions that may not be improperly indulged on this occasion. In the first place, the case furnishes intrinsic evidence that Dr. Cartoni trusted to the symptoms described by the patient, and made no *accurate* examination of the naked body, at the time when he was first consulted. It is quite impossible that between the 25th of October and the 2d November, the disease above described could have formed, or even greatly augmented. In October, all was referred to the spine—in November, an immense tumour was found in the abdomen! The statement that *pressure* on the spinal column did not increase the pain, would infer an actual examination of the naked body—but the history which follows leads to the inevitable conclusion, either that the naked body was not examined—or that only *one side* of the case was investigated. Dr. Cartoni does not seem to have remembered the wise maxim—" audi alteram partem." No! In Italy, as in England, the patient's story appears to be heard, with some degree of impatience, and a hasty conclusion is drawn, without any examination of the organs or parts to which the patient refers, as the seat of suffering. What would be thought of a surgeon, who, on being called to a lady, some nine or ten months after her marriage, complaining of pains in her back, should sagely conclude that there was inflammation of the spinal marrow, without making any examination of the abdomen? That Dr. Cartoni would have found the same tumour in the abdomen, on the 25th October, which he afterwards found on the 2d of November, (had an examination been made,) will not be denied by any man in this world, who knows an atom of the nature and progress of organic diseases. The surprise, therefore, which he evinced, on discovering the tumour, should have been mixed with no inconsiderable degree of shame;—and we hope that this example will not be lost on the rising—and even the falling generation of the profession.

In the second place, we object to the term *abscess* being applied to this disease. It was a large hydatid, within which a number of other hydatids were produced —and the fluid, said to be purulent, fetid, flocculent, &c. &c. was nothing more than a modification of the fluid usually found in hydatid cysts. We hope this case will make the proper impression on medical men, and help them to believe that the investigation of diseases requires all the aid of all our senses—and that he who scouts any auxiliary that does not come within the range of common *routinism*, is an enemy to the progress of medical science, and to the honour and respectability of its professors.

### 47. WALKING THE HOSPITALS.

———viresque acquirit *eundo*.—*Virg.*

In a late Number of the Medical Gazette, there was a letter from " a Bartholomew Student," on what is commonly called walking, or, more properly speaking, *running* the hospitals. In the letter in question, the gentleman states, what is perfectly true, that, be the present system as bad as it may, and in many instances undoubtedly is, still more, after all, depends on the pupil himself than on his teacher. It has been said that the administration of an oath in courts of justice is a mere dead letter, because an honest man will tell the truth without it, and a rogue will not regard it. It must be remembered, however, that there is a very large class neither strictly honest, nor downright rogues, who might be tempted, by interest or convenience, to *tell* a lie, but would probably shrink from *swearing* one. Precisely the same line of argument might be applied to clinical instruction. If a pupil be exceedingly diligent, he will do almost as well *without* it; and if a pupil is exceedingly idle, he will be little or none the better *with* it. But here again, the majority is made up of young men who are neither very industrious nor the reverse; who might be disposed to work, if the path was smoothed for them; but turn aside in despair, when they find that there are no facilities—no encouragement—in short, that all is to be done by the sweat of their own brow.

We can scarcely conceive a more ludicrous scene than may be witnessed any day in the week, at most of our London Hospitals. The surgeon is announced, calls for his book and his dresser—sees half a dozen bad legs in the out-patient's room, and then proceeds to *walk* the hospital, with a mob of some seventy or eighty pupils at his tail, like so many *sheep* after a shepherd. Now commence the scrambling and *mélée*; some trying, vainly trying, to catch a glympse of the case—others to pick up an observation of the surgeon's, the latter reminding one irresistibly of the lines in Goldsmith—

> "And still they gazed, and still their wonder grew,
> That one small head could carry all he knew."

This is the industrious portion of the crowd; the rest, and by far the greater number, may be seen following in the *wake*; sauntering along with halcyon listlessness of mind, or collected into groups, and discoursing most learnedly on the last new farce, or the comparative merits of Cribb and Scroggins, those "heroes of the ring." For the truth of this picture, ridiculous and overcharged as it may appear, we appeal to all who are in the habit of frequenting our hospitals, though we are willing to confess that, within the last year or two, there has been an improvement manifested on the part of the pupils. This improvement we do not hesitate to ascribe to the impulse which has of late been given to clinical instruction; and we are convinced that if this mode of conveying information was more generally and systematically adopted, the greatest possible benefit would accrue, both to the teacher and the taught. At present, even at those hospitals where clinical lectures *are* delivered, they are squeezed out in such a costive manner, as to

> "Keep the word of promise to the ear,
> But break it to the hope."

We would recommend the physicians and surgeons of our public institutions not only to give, but to give freely—not to content themselves with a clinical lecture once a month, or once a fortnight—not to think that when they have done this, they have done much, but that much is to be done—not to sit, wrapt up in imaginary dignity, upon their hill, but to put their shoulders lustily to the wheel, for the sake of science, and, what is more, for their *own* sakes. "To your tents, O Israel," say we, and we trust that "Israel" will take the hint. Let each of the medical officers attached to the hospital have his clinical clerk, chosen from amongst the most diligent and best-informed of the pupils; let this clinical clerk be allowed every facility in the taking and recording of cases, and be responsible for their accuracy; and, above all, let these authentic records of hospital practice be open, *at all times*, to the inspection of the pupils. Upon the most interesting cases, and, of course, only those possessing some interest ought to be recorded in this manner, clinical lectures should be delivered. Now we believe that, at most of the hospitals, (we know that it is so with some of those at the West End,) there are two days in the week set apart for the reception of new patients, and on which the medical attendants are not in the habit of going their rounds within the house. The business, on these days, is soon got over, and they should be devoted to the clinical lecture, which need seldom last more than half an hour. If, then, two of the medical officers will dedicate, each half an hour, once a week, to clinical instruction, it will surely be no great tax upon their time, and will prove of the greatest advantage to their pupils, as well as ultimately to themselves. If two lectures in the week are found to be too much during the Winter season, (we are not of that opinion ourselves,) let only one be given, and the number increased during the Summer; but, in God's name, let something be done, and that quickly, to infuse a little zeal and energy into the minds of our medical students, very many of whom, at present, "crawl from the cradle to the grave" without a particle of either one or the other.

---

48. CHRONIC ULCERATIONS OF THE TONGUE AND PHARYNX CURED BY IODINE. By M. MAGENDIE, Physician to the Salpétrière.

The illustrious author observes that he never enters this melancholy asylum of reputed incurable diseases (the Salpétrière) without turning his mind to the search after a remedy for some of the terrible afflictions which surround him.

The cases of cancer are above all deplorable, and he is not without hope that the large doses of iodine which he is now employing in this disease, may prove serviceable. In the mean time, he reports two cases of old ulcerations considered incurable, which have completely yielded to the remedy in question.

*Case* 1. A female in the ward St. Nicolas, of lymphatic constitution, had enjoyed good health till the age of 30, when menstruation became irregular, and epileptiform attacks made their appearance. After a time, a new malady supervened. Ulcers of a kind which the patient could not well describe broke out on various parts of the body and limbs:—some exfoliations of the tibiæ and bones of the arm also took place. Excrescences were now seen on the pharynx and tongue, and the attendant physician, conceiving the complaint to be syphilitic, notwithstanding the positive denial of the woman, she was put upon a mercurial course. Under this treatment the ulcerations of the body and limbs healed; but those of the tongue increased. In the course of time the patient lost her voice, which was attributed to ulceration of the chordæ vocales. In this deplorable condition, M. Magendie ordered a solution of the hydriodate of potash to be exhibited, and the dose to be gradually increased, till it amounted to 36 drops in the day. The good effects were soon conspicuous. The surface of the ulcerations cleaned, and, in fifteen days, those of the tongue were completely healed. In a little more than a month, the other ulcers were also cicatrised. When every thing appeared to promise success a violent dyspnœa came on, and all the symptoms usually attendant on œdema of the glottis. Antiphlogistics failed, and tracheotomy was not practised. The patient sank. On examination, the interior of the larynx was found covered with firm and whitish excrescences, ("vegetations,") by which the passage was rendered impervious to the air.

*Case* 2. Madeline Petibon, aged 41 years, had been in the Hôpital Saint Louis four years previously for large ulcerations on the legs. She had scarcely left the hospital, apparently cured, when she was seized with dyspnœa, pain in the region of the larynx, and complete loss of voice. These symptoms continued, and, at the same time, large ulcers broke out on the face and neck, as well as on the tongue. Various modes of treatment had been put in practice, but without much relief, and she entered the Infirmary of the Salpêtrière, in March 1827, three years after the commencement of the facial ulcerations. Her nose was now almost demolished—various fungous ulcers were spread over the face and tongue—deglutition was very difficult—the respiration was impeded—articulation almost annihilated. On the 27th June the patient was put on the use of tincture of iodine, and the dose was gradually increased. The ulcerations at last were entirely healed, and a complete cure is now effected.—*Journal de Physiologie.*

---

49. Wound of the Anterior Tibial Artery.

The following case is interesting, as it shows how soon a retrograde current of blood may be established in arteries, and hæmorrhage thus produced in divided vessels, unless a ligature be applied *below* as well as above a wound. The case occurred in the practice of Dr. R. N. Smith, now Professor of Surgery in the University of Maryland, and successor to Professor Granville Sharpe Pattison, of the London University.

"The patient, a Mr. Blodget of Jericho, Vermont, wounded the anterior tibial artery, a little below the middle of the leg, with a narrow chisel which entered from the outside of the leg, just anterior to the fibula, and divided many of the fasciculi of the exterior muscles which envelope the artery. Undoubtedly the artery was merely partially divided by the angle of the instrument. I infer this from the effects which resulted.

"At the instant of the wound there took place a copious gush of blood, which very much alarmed the patient and friends. Dr. Hamilton, an intelligent young physician, was sent for, who, on his arrival, found no difficulty in suppressing the hemorrhage for the time by means of compression. As he had reason to believe the artery completely divided by the instrument, he presumed that the pressure being continued, the hemorrhage would not recur. The patient continued apparently to do well, no more than the usual degree of inflammation supervening, until the end of two weeks, at which time, on attempting to make a greater effort than usual with the limb, blood gushed from the wound as furiously as when the limb was first wounded. Dr. H. was immediately called, and on his arrival the hemorrhage was commanded as before by employing pressure. The limb being at this time a good deal tumid, from diffusion of blood in the cellular tissue, Dr. H. presumed that the artery could not be found by searching the wound.

" After this the bleeding occurred at intervals for two or three days, when I was called to the patient at Dr. Hamilton's request. We agreed that, as the wound was remote from anastomosing branches, and as the vicinity of the wound was much injected with blood, the best method would be to cut for the artery above the wound, and very near the middle of the leg. I immediately proceeded to the operation, and without much difficulty exposed the artery where it lies deeply imbedded between the tibialis anticus and flexor communis muscles. A ligature was thrown round it, not without some embarrassment, in consequence of its depth and the tension and volume of the muscles. Before tying the ligature I held my finger upon the artery, and distinctly felt its pulsations, and in order to assure myself that the artery was included, I drew upon the two ends of the thread, and immediately the artery ceased to beat. This I repeated several times, so that there could be no doubt that the artery was secured. The ligature was then firmly tied, and immediately the pulsation, which before could be seen in the orifice of the wound, ceased entirely, and the blood also ceased to flow.

" As the hæmorrhage did not return, I left the house with very little apprehension as to the result. As I was many miles from home, however, I slept that night in the neighbourhood. In the morning I was much surprised at being called in haste to visit the patient on account of the return of the bleeding. On my arrival, however, it had again ceased; yet there was obviously a pulsation in the wound, similar to that which had been observed before the operation.

" On applying my finger to the anterior tibial, upon the instep, (where all pulsation had ceased immediately after tying the ligature) I was surprized to find that the artery pulsated vigorously, and on comparing its beat with that of the opposite foot, I was still more surprised to find that it was much stronger and more full in the wounded limb than in the other. To ascertain the direction of its current, I then placed one finger upon the upper part of the artery, and pressing it firmly so as to stop its circulation, I felt for its pulsations below, and found them still vigorous. I then reversed this, and found that the blood was passing in a full stream in the retrograde direction.

" I thought it advisable, before attempting any thing further with the knife, to endeavour to prevent the recurrence of bleeding by compressing the artery upon the top of the foot. This was accordingly done, and apparently with good effect. As, however, I could not remain with the patient to witness the result, I advised Dr. Hamilton, should the bleeding recur, to cut down upon the region of the wounded artery, and endeavour to make compression upon the wounded vessel. This he was subsequently compelled to do, and with difficulty he saved the limb."*

---

50. THE INFLUENCE OF ANATOMICAL VARIETIES ON SURGICAL OPERATIONS. By M. ROBERT.

Anatomical deviations have been studied physiologically, and from this study have been deduced the laws of their formation, and the development of monstrosities. The study of these varieties of structure, under a chirurgical point of view, would be of great benefit to the surgical world, and to society at large. Suppose, for example, a surgeon is called on to operate for an aneurism of the brachial artery at the bend of the arm,

---

* Philadelphia Monthly Journal, No. I. Vol. II.

and is ignorant that this vessel may bifurcate as high up as the axilla. Instead of searching carefully for the vessel which is diseased, he ties that one which lies in the usual place. Meanwhile the tumour enlarges, and at length bursts. Attention to the study of anatomical varieties prevents such oversights as these. It is of great importance that the surgeon, when operating, should be acquainted with all the anomalies of structure which the part may possibly present. With this knowledge in his mind, he will not be alarmed or embarrassed in his operation, should the anomaly exist. M. Robert has evinced great industry and research in this extended Essay, and the order which he follows in his inquiries, consists in a successive examination of the great regions of the trunk and the extremities, subdividing his researches into varieties of structure in the bones, the ligaments, the muscles, the vessels, the nerves—and lastly the viscera themselves. We shall follow our author, and endeavour to present our readers with a succinct account of his researches.

### I. The Head-bones, and Face.

The cranium presents great varieties in structure and shape. We often meet with depressions and protuberances, which might be confounded with sanguineous tumours or fractures of bone, after contusions. The defect of ossification, in consequence of which the fontanelles sometimes remain unclosed, is a common variety—but it is not so well known that the brain sometimes protrudes through these apertures, and forms an *encephalocele*, which becomes strangulated as the bones thicken; and requires the trephine at the sides of the unnatural opening. In very old people, the skull becomes so much attenuated, in some places, as to render operations hazardous, if this circumstance be not borne in mind. The frontal bone has remained divided by a prolongation of the sagittal suture, and has been taken for a fracture, of which a remarkable instance is recorded by Quesnay.

A very curious case of development of the vessels of the diploe is given by Frank.

A peasant, aged 24 years, in a scuffle with a soldier, received a sabre wound on the anterior and right side of the frontal bone, an inch and a half from the sagittal suture, where it is obliterated at this place in the adult. The sabre had removed a portion of the external table of the skull. The man neglected himself for some days, and when Frank arrived there were such symptoms as induced him to apply the trephine in the neighbourhood of the wound. As soon as the saw had arrived at the diploe, a torrent of blood issued forth—and, in short, the patient died of the hæmorrhage the same day. On dissection, it was found that there were seven vascular communications between the dura mater and the diploe, through so many foramina in the internal table of the skull, at this place. Hence the fatal hæmorrhage. More recently, Magendie attributes to this kind of hæmorrhage, the majority of those cases reported by writers, where *extravasated* blood has issued forth after the application of the trephine.

A great many varieties occur in the anatomy of the face. The supra-orbital foramen may be placed quite behind the orbit, making it extremely difficult to divide the frontal branch of the fifth pair, in the tic douloureux. The os unguis is sometimes wanting, and its place supplied by the nasal process of the superior maxillary bone. In such a case considerable difficulty would be experienced in perforating the bone for fistula lachrymalis. In some individuals a middle bone is found between the two portions of the inferior maxillary; it may either be single or divided by the symphysis into two, and, from its mobility, has been mistaken for a fracture of the lower jaw and treated accordingly. There may be two ranine arteries, so that in glossitis our author prefers making deep incisions on the dorsum of the tongue, to opening the ranine veins. When, as sometimes happens, the membrana pupillaris remains after birth, it is necessary either simply to puncture it, make a free division, or cut away a point of the circumference of the iris, and form an artificial pupil. The inferior orifice of the lachrymal canal is frequently guarded by a valve, obstructing the passage of the stilet. There are sometimes two stenonian ducts which should be remembered in the operation for salivary fistula. Not unfrequently the spinous process of the seventh cervical vertebra, is separate from the rest of the bone and moveable beneath the skin. After an injury upon the part, this might readily be mistaken for a fracture. The scalenus medius of Soemmering, when it exists, separates into two bundles the nerves of the axillary plexus. This would cause much embarrassment in the operation for tying the subclavian. Sometimes the muscle is directly interposed between the artery and nerves which would completely protect the latter, and in a child, which we had an opportunity of examining last Summer, there was no scalenus *anticus* muscle at all, so that the subclavian vein lay in contact with the subclavian artery. The omo-hyoideus may arise not from the scapula, but from the middle of the clavicle, which would also embarrass the operator on the subclavian.

### II. Vessels of the Neck.

Allan Burns mentions an instance, and Harrison, we believe, gives another, where the *right* subclavian arose from the *descending* arch of the aorta, and passed across the neck in front of the trachea, as high as the inferior border of the thyroid gland. In such a patient, were tracheotomy performed, the artery would most inevitably be wounded. It must have been some irregularity in the distribution of the great vessels which happened to a celebrated professor of surgery at Berlin, who having performed the operation of bronchotomy on the daughter of a brother-professor, the patient died under his hands.* The subclavian may take the same origin but pass behind, instead of before the trachea, between it and the œsophagus. In such a case, care would be required in performing œsophagotomy. A third variety in the course of the right subclavian, is where it takes its rise from the arch of the aorta, beyond the origin of the left subclavian, and on the left side of the body of the second dorsal vertebra. It then crosses in front of the spine, and behind the thoracic duct and œsophagus, lying much exposed to wounds from foreign bodies sticking in that tube. In 1815, Mr. Kirby* was summoned to a woman, who had swallowed a piece of bone, and who, in spite of tracheotomy, died of suffocation and hæmorrhage, apparently from the interior of the œsophagus. On dissection, it was found that a spicula of bone had pierced the posterior wall of the œsophagus, and transfixed the right subclavian, which took this unusual course. When there is this variety, the nervus vagus on the right side, occasionally sends off no recurrent branch, its place being supplied by several branches arising from the inside of the vagal trunk. In such a case, if the carotid were tied these branches would probably be cut, and the voice perceptibly affected. Immediately after its exit from between the scaleni, the subclavian, in some instances, becomes fairly enveloped in the brachial plexus. Perhaps it was owing to this anomaly that the nerve was tied instead of the subclavian, in the case related by Sir Astley Cooper. Zagorsky has observed, that the innominata on the right side was entirely awanting, but it existed on the left.†

It has been well remarked by Hodgson, that aneurism of the innominata, or even of the arch of the aorta, will frequently rise so high in the neck as to be mistaken for aneurism of the carotid, or even of the subclavian. A case of the latter kind is mentioned by Allan Burns, where it was proposed to tie the subclavian artery. Fortunately, however, this operation was not performed.

The right carotid may arise from the arch of the aorta, instead of the innominata. It then mounts up a couple of inches, or more, in front of the sternum, and crosses the trachea, lying exposed, of course, to injury in tracheotomy and wounds of the neck. Both carotids, on the other hand, may arise from the innominata, and then the left takes the course in front of the trachea. In such a case it may admit of doubt whether a ligature of the innominata would not be attended with very serious results in consequence of the stoppage of the flow of blood at

* Walter. Mémoires de l'Académie de Berlin.

* Dublin Hosp. Reports, v. iii. p. 224.

† The innominata has also been seen rising so high in the neck as to be endangered by the razor of the suicide, or the knife of the tracheotomist.

the same instant through one subclavian and both carotids. Portal has related a case in which extirpation of an enlarged tonsil proved fatal in consequence, it would seem, of a wound of the internal carotid, which lay extremely near it. Barclay, also, mentions a similar case.

Allan Burns observed in a child, that the left vertebral artery took its rise from the arch of the aorta, whilst the right arose from the subclavian, and passed up behind the carotid, along with the sympathetic nerve as high as the third cervical vertebra, where it entered the osseous canal in the transverse processes. In such a case, there would be some danger of including this artery in the ligature if it were necessary to tie the carotid, and besides, an aneurism of the artery in this situation would be readily confounded with one of the carotid itself. However, if a surgeon follows the excellent rule of never drawing the knot upon a vessel till he is satisfied that pressure of it between his finger and thumb stops the flow of blood into the sac, this blunder can never happen. Some varieties occur in the distribution of the thyroid arteries which we shall briefly notice. The superior may arise from the common carotid low in the neck, pass up alongside of that vessel, and then when it has arrived at near the angle of the jaw turn downwards to the thyroid gland. In this variety the vessel is large, and would be in the way both of the external incisions and the ligature in the operation on the carotid trunk. The inferior thyroid also, having taken its usual origin may pass directly in front of the common carotid below. Burns saw both inferior thyroids arise by a common trunk from the subclavian or aorta, which would add to the danger of œsophagotomy. In tracheotomy, it should be remembered, that there is very frequently indeed a large artery, called by Harrison the thyroideus medius, which arises from the common carotid, and passes in front of the trachea. Burns mentions a case where this artery arose from the innominata, and passed upon the œsophagus as high as the left lobe of the thyroid gland. The patient was a young lad, and œsophagotomy having been performed, the bleeding from the vessel was so considerable as to require a ligature.

In the operations on the neck, the veins are often sadly in the way of the surgeon's knife. There may be two or even three external jugulars on one or both sides. Another variety has been noticed by Wilde, where a vein as large as the jugular, was formed by the union of two branches in front of the trachea, and proceeding upwards from thence dipped down by the side of the thyroid gland to join the internal jugular. Of course in laryngotomy and tracheotomy, such a distribution might be productive of considerable embarrassment. A case is related by Virgili in the Memoirs of the Academy of Surgery, where, having opened the trachea between the rings, such hæmorrhage took place, that the blood getting into the trachea excited a most violent paroxysm of coughing, forcing out the canula whenever it was introduced. In this dilemma, the man being almost suffocated, Virgili cut longitudinally into the trachea down to its sixth ring, and held the patient's head out of bed, with his face towards the ground. This operation was perfectly successful. M. Roux, in a similar case, introduced a silver catheter into the trachea, and sucked out the blood.

The subclavian artery, external to the scalenus, where it is usually tied in operations, is described as lying in a triangular space, the base of which is formed by the subclavian vein, and the sides by the scaleni muscles, and axillary plexus. The vein however, is sometimes placed much higher than it *should* be. A man had a large aneurism of the subclavian, and the operation for tying this vessel was performed in the usual situation. The tumour was a good deal in the way, but the operator at last arrived at what he considered was the artery, and he was confirmed in this opinion, by finding that, on raising the vessel, the pulsation in the tumour ceased. The ligature was applied—bad symptoms supervened—and the patient died. On dissection, it was discovered that one of the brachial nerves and a *part* of the subclavian vein had been tied, the needle having passed *through* the latter vessel, which lay higher in the neck than usual. The cessation of pulsation in the sac, on lifting up the nerve was explained, by its having dragged up the artery with it so as to produce considerable compression. The subclavian vein has likewise been seen passing *with* the artery, *between* the scaleni; in this variety it might either be tied for the artery, or both included in the one ligature. Morgagni found two subclavian veins, uniting where they joined the internal jugular, which would also be an awkward anomaly.

### III. VARIETIES IN THE ANATOMY OF THE THORAX.

These are few. The last rib may be deficient, with or without deficiency of the corresponding vertebra. An intercostal space will then be wanting, which should be recollected in the operation for empyema. Two ribs may be joined before or behind, or they may be entirely joined together, and then the double rib is commonly inserted into the sternum by two cartilages. Many varieties occur in the location of the muscles, but these we shall pass over as they are of little practical importance. There may be two intercostal arteries, one taking the course of the mammaria interna, the other lying in the middle of the intercostal space, and exposed in the operation for empyema. In a phthisical patient opened by Morgagni, death was occasioned by rupture of the vena azygos which was as large as the superior cava.

This concludes the first part of M. Robert's Memoir, and we freely award him our tribute of approbation for the patient industry in collecting facts which it evinces. Such men are the pioneers of science, and their task, though uninviting and laborious, is of infinite importance. The subject of surgical anatomy is scarcely paid sufficient attention to in this country, or if it were we should have fewer opportunities of seeing reports of errors and unfortunate operations blazoned forth before the gaze of a credulous and greedy public. We have been at considerable pains to detail the varieties which occur in the vessels of the neck, as the anatomy of that truly *surgical* region is complicated at the best, and the blunders which have been made in it, even lately, are unluckily notorious. In a succeeding number of the JOURNAL DES PROGRÉS, we are promised a continuation of the Memoir, and we shall take care to lay an abstract of it, when it comes, before our readers.

### 51. HALLUCINATIONS TREATED WITH COLD WATER. By M. F. BROUSSAIS.

A soldier, of the 30th regiment of Light Infantry, drank some brandy early in the morning of the first of January, 1828—and breakfasted afterwards, at 10 o'clock, in his usual manner. At 12 o'clock he went to sleep, and was roused up at one o'clock to go on guard. At four o'clock, he and two of his comrades drank three bottles of wine at dinner, without feeling or evincing any disorder. At six o'clock he felt a general sense of cold, with horripilation and tremor in all his limbs, while, at the same time, he gave vent to loud lamentations and shed some tears. These symptoms were succeeded by great heat, restlessness, vertigo, and ultimately by convulsions. He was taken up by his companions, and carried to his bed; but he there became furious and ungovernable, requiring seven or eight people to confine him, and being quite incapable of recognizing his comrades. Instead of these he appeared to behold Satan and a legion of devils around him! At ten o'clock at night, M. Broussais arrived, and found the patient confined partly by cords, and partly by the hands of his companions. He seemed busily engaged with the Satanic bands that attended him, and his countenance had something horrible, and, at the same time, ludicrous in its expression. But the devils were not his only tormentors. A young woman, to whom he had paid his addresses, was present, (in his imagination,) coquetting with certain of his rivals, which appeared to annoy him as much as his infernal visiters. M. Broussais applied cold water to the surface of the body, and caused the patient to swallow a pretty considerable quantity of this cooling beverage, for the space of two hours. This procedure had a surprising effect. The devils began to retreat, in dancing attitudes, which caused the soldier to break out, at last, in loud laughter, at the ridiculous figures which his enemies cut. His sweetheart, too, entirely altered her conduct, and harmony was restored.

Amantium iræ redintegratio est amoris.

By one o'clock in the morning, the hallucinations were pretty well dispelled—he recognized his SERGEANT—and the sad realities of this dull world were once more substituted for the vivid scenes of a distempered *imagination!* We say *imagination*—for it would be sad heterodoxy to impute these hallucinations to a *disordered brain* or other material organ. The cold water and cold drink acted, no doubt, on that "divinæ particula auræ" of our composition, and in this way cured the effects of the brandy in the morning and the bottle of wine at dinner, which seem to have conjured up "in the mind's eye" a strange assemblage of carnate and incarnate devils.

There was a period when JORDY (for this was the soldier's name) would have been pronounced to be "POSSESSED"—not of brandy and wine—but of a veritable DEMON. Our philosophy is a little changed. Some of our physicians (the *immaterial* ones) now pronounce these hallucinations to be "*mental* diseases"—not *corporeal* disorders. These philosophic physicians seem to forget that they are the real advocates of materialism—and that, in imputing to *soul* the liability to disease, they necessarily subject it to the consequence of disease—DEATH!

### 52. ILLUSTRATIONS OF THE ORGAN OF HEARING.*

The author of this Essay has for a long time devoted a considerable share of his attention to diseases of the ear. His "Illustrations of Acoustic Surgery" were very favourably noticed by many critics, and stamp him as a man fully capable of taking advantage of the practical suggestions of others, and of adding much from the resources of his own mind. From having seen a great number of cases of diminution of hearing which arose from imperfect secretion of cerumen, he has naturally been induced to reflect carefully on the probable uses of this secretion, and its effects on auditory perception. In order to form as correct ideas as possible on the subject, he has consulted various works on acoustic surgery, but the substance of the information he was thus able to glean consisted merely in the observation, "that deafness sometimes depended on a defective secretion of the cerumen, and a consequent dryness in the meatus." Why a deficiency of cerumen should diminish the power of hearing had not been determined; he, therefore, commenced a series of experiments, the result of which has been the production of the pages we are now to notice. The bitter taste and glutinous consistence of the cerumen, so well adapted, in most instances, to protect the tube and internal parts of the organ from the intrusion of insects, has attracted the attention of various writers. But the author hopes that he has succeeded, in the present volume, in more fully explaining, than has hitherto been done, the beautiful arrangement by which this secretion is rendered subservient *to*, and, at the same time, combines *with*, and produces other and more comprehensive effects on auditory sensation.

Mr. Buchanan has carefully examined the glands which secrete the cerumen, and has suggested some valuable practical observations upon the importance of their perfect and healthy action. We find, upon referring to several modern works on anatomy and physiology, that this subject has been scarcely touched upon. Mr. B. states that, "owing to the immense number of these glands, they are situated so close to one another, that the integuments on which the ducts open appear like a sieve. The mouth of one duct almost touching that of another, and when they deposit the secretion uniformly from the ducts on the surface of the auditory tube, a *ceruminous tubular circle* is formed, that causes a contraction of the meatus." When the undulations of sound reach the ceruminous tubular circle, they are thrown towards the centre of the tube, by means of this secretion, which contracts its caliber. In their progress through the tubular circle the asperities of the pulsations are softened by its chemical properties, rendered melodious and congenial to the perception,

* Physiological Illustrations of the Organ of Hearing, more particularly of the Secretion of Cerumen, and its Effects in rendering Auditory Perception accurate and acute; with further Remarks on the Treatment of Diminution of Hearing, arising from imperfect Secretion, &c. being a Sequel to the Guide, and to the Illustrations of Acoustic Surgery. By THOMAS BUCHANAN, C.M. &c. &c. &c. Royal 8vo, pp. 160, with plates. 1828.

at the same time condensed, and also strengthened by means of the ceruminous tubular circle which contracts the contracting meatus, and in this manner the current of pulsations reaches the membrana tympani in regular undulatory succession. These ingenious physiological views furnish our author with many practical hints, from which he has derived much advantage in the alleviation of different species of partial deafness, arising from the diminished secretion of the cerumen. Mr. Buchanan touches rather sarcastically upon the frequency of the operation of puncturing the membrana tympani, and upon the idea "that the loss of the tympanum itself is not so detrimental to hearing as it is generally believed!"

According to this grade of the march of intellect, he observes, that we ought not to be astonished if it were to be asserted, that the whole of the osseous parts of the organ are not necessary to hearing, only the portio mollis. Such an assertion has been made, and cases in point published,* one of which we will briefly relate, for the satisfaction of our author, as well as our readers. A young girl, at the termination of severe scarlet fever, was affected with violent inflammation, followed by suppuration of the internal organ of hearing. Caries of the bones followed, and the malleus, stapes, and incus were discharged. The tympanum was also destroyed. The power of hearing, however, was not diminished. Gräfe, one of the editors of the able German periodical, from which we have taken the heads of this curious case, afterwards saw the patient who had been attended by Dr. Wolf. A similar case is related in the Phil. Trans.

Mr. Buchanan states, that however elegant may be the shape, and however exquisite the organization of the ear, it will be of comparatively little use to the possessor, unless the tube be lubricated by healthy cerumen, which modifies the undulations, otherwise irregular and indistinct, and transforms them into audible and accurate sounds. About three years ago he presented to the Royal Medical Society of Edinburgh, a specimen of the olfactory nerves of the human subject. He performed the dissection in the course of an hour. The preparation is allowed to be the best of the kind, and caused much interest at the time, from the rarity of such specimens of delicate dissection. The author makes some interesting remarks on a species of diminution of hearing, which he believes not to have been noticed by any previous writer. This form of disease is said to depend upon the extraordinary growth of the pili auriculares, which sometimes increase so much in number and size as to impede the power of hearing, and thus a particular species of disease is produced, termed by Mr. Buchanan *hirsutus tubulus*. "It consists of an almost innumerable quantity of hairs that shoot across and block up the tube which is in general completely dry and destitute of secretion." The author has seen many patients afflicted with the *hirsutus tubulus*, and he discusses at some length, the nature of the disease, and the treatment he proposes for its relief. He also details some cases which he successfully treated. It has been supposed, that cold produces an unusual secretion of cerumen, and that the meatus is thus occasionally blocked up. According to the observations of the author, however, the consequence of cold, when affecting the ear, is to *suppress* the secretion of the cerumen.

Several very important criteria are to be found in the shape and size of the auricle, especially in the angle which it forms with the cranium, and also in the size and depth of the concha, which may assist to determine the prognosis in cases of diminished hearing when the symptoms are obscure. Mr. B lays down several axioms respecting the figure and aspect of the part, as practical guides for the aurist. He observes, that females ought to be particularly careful in the form of their head dress, so that it may not interfere with, or bind the auricle flat to the head, as the continuance of this kind of pressure, will sooner or later, cause the cartilage of the auricle to contract the horizontal diameter of the meatus, and ultimately block it up.

An Appendix is added, containing physiological and anatomical remarks on the structure of the organ of hearing in different animals. There are also many

* Journal für Chirurgie, und Augenheilkunde un Gräfe und Walther. Bund, 7. Stück, 2. 299.

neatly executed plates, in illustration of these observations. Upon several hitherto neglected points, relative to the structure and diseases of the organ of hearing, Mr. Buchanan has made many original and important additions. His work cannot, therefore, be consulted without advantage by those who feel interested in the subjects upon which he treats.

---

53. CASE OF OBSTINATE COUGH CAUSED BY ELONGATION OF THE UVULA, AND CURED BY EXCISION OF A PART OF THE SAME. By Dr. PHYSICK, Professor of Anatomy in the University of Pensylvania.

Relaxed and elongated uvula is much more frequently a cause of troublesome and harrassing cough than is generally imagined. Next to examination of the chest, the state of the throat should be investigated. Where the cough is dry and very frequent, without febrile symptoms, we may suspect elongated uvula, and, at all events we should inspect the fauces. The following very curious case recorded by Dr. Physick, is well deserving of attention.* The patient was a young lady who had come from New Orleans to consult Dr. P. for an obstinate cough. The history of the complaint is thus stated.

" 'The first circumstances which had any connexion with the singular affection of this young lady, were a complaint of constant head-ache, attended with a disposition to vomit without nausea occurring first, during convalescence from an attack of remitting fever, in the middle of May, 1826. The latter symptom soon became the most prominent, and increased to a constant effort to retch, in which nothing was thrown up from the stomach, and which was not relieved by free vomiting. At this time no complaint of pain was made any where but in the head.

" 'Considering the gastric irritation as sympathetic of an incipient cephalic affection, leeches were applied to the temples and behind the ears, and some doses of active cathartic medicines given. No advantage was derived. The retchings became nearly constant, and from a noisy effort to vomit, it gradually changed to a convulsive cough, altogether involuntary and uncontrollable, and conveying an impression as if something obstructed and irritated the organs of respiration. This is, as nearly as it can be described, the character of the cough ever since.

" 'The first paroxysm increased in violence for a number of days, and until the 8th of September, when, about midday, after vomiting, (which was at this time not unusual with her,) in which she threw off a quantity of white tough mucus, she fell into a state of extreme prostration. The cough ceased and she appeared to be dying. From this she slowly revived through the evening, and on the next day there was a degree of reaction amounting to fever, which gradually subsided and left her quite well.

" 'The mucous expectoration, likewise, though at the time regarded with some interest, has, in the latter attacks, been produced occasionally in vomiting, but never followed by the same alleviation. On the recovery from the first attack, she remained well for two weeks, when she was again seized with the same spasmodic cough, attended with pain in the breast, but not preceded as before with any irritation of the stomach. This, after continually increasing in violence for about eight days, again left her in nearly the same manner it had done in the first instance. After an interval of three weeks, she had another attack of the same duration, and of extreme severity. Since this there has been two more, but at longer intervals, and not altogether of the same severity.

" 'The dates of the different paroxysms are the early part of September—of October—of November—of January—and of May. During the long interval between January and May, a slight cough of the same peculiar character has seized her every morning on awaking, after which she remains entirely exempt for the remaining twenty-four hours. At first it lasted for a few seconds only, but its duration gradually increased to thirty or forty minutes. Since the last violent attack it has been reduced to only a few moments continuance.' "

Various remedies being tried without benefit, the lady was sent to Philadelphia.

---

* American Journal of the Medical Sciences, February, 1828.

Dr. Physick immediately suspected the cause of the disease, and, on examining the throat, he found an elongation of the uvula. The excision was performed, "immediately after which all the symptoms entirely ceased, and have not since returned in the slightest degree."

Dr. Physick was formerly in the habit of using scissors for the removal of elongated uvula, but has recently invented an instrument which renders the operation more easy. We cannot describe this instrument in words.

---

### 54. PURITY OF SULPHATE OF QUININE.

The importance of this medicine is now universally acknowledged; but, unfortunately, its great price presents perpetual temptation to adulteration. Detection is our only remedy—for if we trust to men's honesty, we shall lean on a broken reed. The following rules for ascertaining the purity or the adulteration of this invaluable medicinal agent, have been published in a recent number of the Philosophical Magazine, by Mr. R. Phillips.

"Pure sulphate of quina has the form of minute fibrous crystals, it is inodorous, and its taste is bitter. If certain vegetable products, such as starch or sugar, be mechanically mixed with it, they may possibly be observed by merely inspecting the preparation with a glass.

1st. If the sulphate of quina be mixed with a considerable proportion of foreign matter, it may probably be detected by dissolving the salt in question in about three hundred times its weight of water,—say one grain in about five fluid drams of boiling distilled water. On cooling, pure sulphate of quina will be deposited in feathery crystals in twenty-four hours, if there be no adulteration.

2dly. As indirect, but as good collateral evidence, the taste of sulphate of quina of known good quality may be compared with that of another sample. Thus, when pure, a grain of sulphate of quina will render nearly a pound and a half of water, or 10,500 grains, sensibly bitter.

3dly. The alkalies, either pure or their carbonates, if but slightly in excess, always occasion precipitation at ordinary temperatures in a solution of sulphate of quina containing only 1-1000dth of its weight, or less than one grain in two fluid ounces of water.

4thly. A solution of tannin occasions a very sensible precipitate in an aqueous solution of sulphate of quina, containing only 1-10,000dth of its weight of the salt, provided there be no acid in excess. Kino is that form of tannin which best answers the purpose. It is however to be observed, that the salts of morphia, cinchonia, strychnia, &c. are similarly affected by tannin; but they are not likely to be mixed with sulphate of quina.

"5thly. Sulphate of quina suspected to contain sugar, gum, or other substances soluble in cold water, may be tried by digesting the same portion of the salt in small and successive portions of water to saturation. If the sulphate of quina be pure, and the solutions all properly saturated, they will have the same taste and specific gravity; and similar portions will yield, by evaporation, equal quantities of solid residuum.

6thly. A repetition of the above process, substituting alcohol for water, answers for extracting resin and some other substances, because sulphate of quina is soluble in alcohol to only a limited extent.

7thly. If a white substance insoluble in cold water be found in the sulphate of quina, heat the mixture to about 170° of Fahrenheit. This will render starch soluble, and its presence may be determined by the addition of an aqueous solution of iodine, which will immediately occasion a blue colour, and eventually a blue precipitate. The iodine should be added in very small quantity.

8thly. Sulphate of quina has been adulterated with ammoniacal salts. These are rendered obvious by adding a little of the suspected salt to a solution of potash. If any ammoniacal salt be present, ammoniacal gas will be readily detected, either by the smell, or by holding over the mixture a piece of turmeric paper, or a bit of glass moistened with acetic acid.

9thly. To ascertain whether sulphate of quina contains any earthy salts, such as sulphate of magnesia or sulphate of lime; burn a portion of it in a silver or platina crucible, or even in a clean tobacco-pipe. Any earthy salt, or any matter indestructible by heat, will of course remain in the vessel.

10thly. To ascertain that the sulphate of quina contains the proper quantity of sulphuric acid and quina, dissolve a little in pure muriatic or nitric acid, and add a solution of muriate or nitrate of barytes: 60 parts should give about 17.3 to 17.4 of sulphate of barytes; or the method may be varied without the trouble of drying the precipitate. Dissolve 60 grains of sulphate of quina in water slightly acidulated with muriatic or nitric acid; add a solution of 18 grains of nitrate of barytes, and separate the precipitated sulphate of barytes by filtering. If nitrate of barytes be now added to the clear solution, it should still occasion slight precipitation, for 60 of sulphate of quina contain 5.8 gr. of sulphuric acid, equivalent to 19.1 of nitrate of barytes.

This test is only to determine that there is no crystallized vegetable matter uncombined with sulphuric acid in the sulphate of quina; the detection of earthy or alkaline sulphates has already been provided for.

11thly. Sulphate of quina should lose not more than from 8 to 10 per cent. of water by being heated till deprived of its water of crystallization. Mr. Barry informs me that he once examined a sample which contained more than 40 per cent. of water in excess diffused through it."

P.S. LIQUOR OPII ACETATIS.—The following formula is given by Mr. Houlton for preparing a solution of opium, equal, if not superior, to the black-drop, and other secret preparations of the same medicine. Five minims of the solution are said to be equal to a grain of opium.

Take of Beaufoy's strong acid ℥j.
 Distilled water .... ℥ix.
 Opium ............. ℥ijss.

Macerate with a gentle heat for five days, and strain.—MED. REPOS.

---

55. LOBELIA INFLATA.—CURE FOR ASTHMA.

This herb grows plentifully in all parts of the United States, and the American physicians consider it as yielding to no plant in powerful and unequivocal operation on the human frame. It possesses emetic, sudorific, and great expectorant properties. We are informed by Dr. Barton, in his "Vegetable Materia Medica of the United States," that the lobelia is chiefly remarkable for its emetic qualities, emptying the stomach vehemently and speedily—producing, however, great relaxation, debility, and perspiration. Spasmodic asthma, hooping-cough, and spasmodic croup, are the diseases in which the American practitioners have found it most useful. From the activity of this medicine—or, in other words, its powerful poisonous qualities, it should be given with caution, and in the doses to be presently described.

Dr. John Andrew has introduced this medicine to the attention of the British profession, through the medium of our Glasgow contemporary, for May of the present year, and the manner in which he first became acquainted with its anti-asthmatic properties is rather singular. Being in a steam-boat on one of the lakes of Scotland, a man was seized with a violent paroxysm of asthma just as the vessel got under weigh. The Doctor was on board—but he had no medicines—and as the doctor is always expected to do something, whether he have drugs or not in his possession, some warm gin-toddy was exhibited, but it only produced momentary relief. At this juncture, a gentleman on board, who had himself suffered from asthma, offered Dr. A. some tincture, which he assured him would give relief to the poor man. A tea-spoonful was given every 20 minutes—"and, in less than 40 minutes, the poor fellow was relieved from the distressing paroxysms." The American gentleman now shewed him the prescription, which ran thus:—℞. Fol. lobeliæ inflatæ, ℥ijss. Alcohol. dilut. O.j. Digere per dies quatuordecem et cola. The gentleman above-mentioned made Dr. Andrew a present of some of the plant, which he formed into tincture according to the above formula, and has now been enabled to give the medicine a fair trial. He is convinced that it is of great use in asthma. He has exhibited it in the worst forms of pertussis "with decided advantage—in all relieving, and, with the exception of one, curing the disease." "There is no medicine with which I am acquainted that so effectually relieves the bronchial vessels of their viscid mucus as the lobelia." The tincture, made according to the above form is stronger than that received from the American gentleman, and should be given in doses not exceeding 30 or 40 minims every twenty minutes. Dr. A. has given some of the plant to Mr. Macleod, of Argyll Street, (Glasgow) who can supply medical gentlemen with the tincture.

---

56. On the Secale Cornutum, or Ergot. By Dr. Dewees, Professor of Midwifery in the University of Pennsylvania.

As few obstetrical practitioners can have had such ample experience in the exhibition of this remarkable medicinal agent as Dr. Dewees, we deem it right to make his opinions as widely known as possible in this country. This distinguished Professor has favoured his brethren with an interesting paper on this subject in the second Number of the American Journal of Medical Sciences, (Feb. 1828) from which the following particulars are condensed.

Dr. D. conceives that the action of the ergot on the uterus is different from that of other stimuli, such as opium, oil of cinnamon, volatile alkali, &c.—or the mechanical stimulus of instruments or the hand. These may rouse the alternate contractions of the uterus into temporary, and sometimes successful action; but the tonic, or more permanent, contraction does not remain with any degree of certainty. The ergot, on the other hand, effects an increase of tone in the intervals between the pains, as well as increases the paroxysmal pains themselves. The promptitude of its action is another peculiarity. If it do not manifest an influence in 20 minutes, or half an hour, it utterly fails. Its success is generally in proportion to its promptitude. By many, this sudden action has been attributed to *coincidence*, rather than the effect of the remedy. This may occasionally be the case; but Dr. D. conceives he can readily distinguish the effect of the ergot from the natural efforts of the uterus, by the following signs.

"When ergot has been administered with success, we find the uterine effort not only more quickly repeated, and more powerfully exerted, but these efforts are accompanied with less suffering, than the same apparent exertions of this organ, where not urged by this drug. The woman, when interrogated with respect to her feelings, expresses her sensations by saying she feels "as if every thing were forcing from her;" but at the same time admits, that the pains have not the same character with those she suffered before: indeed it very frequently happens, that there is a great abatement of suffering, by converting a concentrated pain, and this most frequently in the back, to a more generally diffused one over the abdomen; or by obliging the back to participate if the abdomen has been the particular seat of it. At the same time it must be confessed, that the intervals between the contractions are more uncomfortable, as an almost constant nisus is kept up by the excited, or rather, it would seem, the goaded uterus, though the sensation does not amount to pain. Now the presence of the circumstances just noticed, constitutes the peculiarity of the action of the secale cornutum."

Dr. D. affirms that he has "the most firm reliance on the powers of the ergot." He generally administers it in substance, in the dose of 20 grains powdered. He does not believe it will retain its medicinal properties more than a year. It should be kept in a well stopped bottle, and powdered only when wanted. He believes there is no well-authenticated case on record, where the ergot has done mischief to the child. Dr. D. criticises the paper of Dr. Henry Davies, on this subject, and lays down the following rules for the exhibition of the medicine.

"1st. It should never be given before the membranes are ruptured, the os uteri dilated, and the external parts disposed to yield.

"2d. It must not be used so long as the natural pains are efficient, and competent to the end.

"3d. But should they flag, from any cause, it may be given; provided the labour be a natural labour according to our acceptation of the term 'natural labour;' that is, when the head, (if well situated,) the breech, the feet, or the knees, present. For, independently of any accident which may complicate the labour, it is sometimes desirable, for the safety of the child, to hasten it when the natural powers are incompetent to this end.

"4th. And if the labour be accompanied by any such accident as flooding, convulsions, syncope, &c. it may sometimes be employed to great advantage, provided rules 1 and 2 are not violated.

"5th. It may be used very often with much advantage in every kind of premature labour; and at full time, when the placenta is not thrown off, and the uterus is found in a state of atony.

"6th. Where flooding takes place after the rupture of the membranes; the os uteri well dilated; the pains feeble, but the child well situated.

"7th. Where the head of the child has been left in the uterus by being separated from its body.

"8th. Where the uterus is painfully distended by coagula."

Dr. D. is against the exhibition of the ergot, in cases of placental presentations, considering that there is no security against hæmorrhage, except by early delivery. He has derived much advantage from a grain of the ergot given three times a day, in cases of mænorrhagia, where the long continuance of the disease, rather than the immediate excess of the quantity discharged, rendered it important to arrest the flow.

---

57. INSTANCE OF OBLITERATION OF THE AORTA OPPOSITE THE FOURTH DORSAL VERTEBRA. BY PROFESSOR MECKEL.

A Peasant, aged 35 years, previously in good health, robust, and well-made, was, all at once, on the 18th January, seized with a sense of great debility, while carrying a sack of grain to market. He was carried to the hospital immediately. The symptoms of syncope and vertigo were dissipated in a few hours; to which succeeded gastric irritability, pain in the chest, total loss of appetite, bilious vomiting, the pulse remaining little altered. By the 6th day, the patient appeared to be completely cured—got up—and was walking about—but suddenly fell down dead.

*Dissection.* On opening the thorax, the pericardium was observed to be filled with black blood, occasioned by rupture of the right auricle, which was softened in its structure. The aorta ascendens was found to be too much dilated for injection from that point—and, therefore, ligatures were thrown on the left subclavian and carotid arteries, while the tube was fixed in the arteria innominata. The injection was considered to be unsuccessful, and as the subject had been designed for a demonstration, it was thrown aside. On opening the abdomen, afterwards, the vessels were seen injected, as were those of the lower extremities down to the feet. The examination being prosecuted, they found the aorta, immediately below the arterial ligament, reduced to the size of a crow-quill, while a beautiful net-work of vessels was seen between the trunks, going off from the arch of the aorta, and the intercostals of the aorta descendens. The said intercostals were very much enlarged, and had produced grooves in the ribs. From this circumstance it was inferred, that the obliteration of the aorta was an affection of long standing, and could not possibly have dated from the late attack of syncope, six days previously. The man must, therefore, have, not only survived the cause of the obliteration, whatever it was, but lived in good health for many years afterwards. On enquiry, all that could be learnt, was, that this man had been very often ill in his youth; but afterwards had grown up strong and muscular.—*Journal Complementaire.*

MR. SYME'S CASE.

When on the subject of obliteration of arteries, we may here notice a case recently published by Mr. Syme. An unhealthy man, aged 58, had been seized with mortification in the left leg, without any apparent cause, and where nothing but amputation appeared to present any chance of success. The limb was removed above the knee, and, on examination, the popliteal artery was found firmly obstructed by a dense white coagulum, like that lining old aneurisms, and this obstruction continued all the way to its division into the peroneal and tibial arteries, which were also obstructed to the extent of an inch and a half from their origin, beyond which point they were healthy. The obstructed vessel seemed also to be somewhat contracted, particularly about the middle of the popliteal portion, where its coats were thickened. The vein was much thickened, so as to resemble an artery, but not obstructed. The man did well for some days, and half the stump was healed. But the breathing became oppressed, and he died. On dissection, the heart was found so soft and flabby, that the fingers could be easily pushed through its substance. The *right* internal iliac artery was firmly obstructed from its origin, as were the right and left internal iliac veins. Mr. Syme's opinion of the case is, that it was one of acute inflammation of the arteries, similar to the one recorded by Mr. Hodgson.

---

58. Accumulation of Fæces in the large Intestine.

We believe that there is much more mischief produced by the retention of stercoraceous matters in the colon and rectum than is generally supposed. M. Guibert has recently reported a case to the Royal Academy of Medicine, which bears on this subject. A pregnant female experienced a fall, and the succussion was received on the right side of the abdomen. This accident was succeeded by pain, and a sense of weight in the pelvis of that side. An inflammation and abscess now took place in the right mamma, accompanied with considerable fever, during which a premature delivery took place. The fever continued after delivery, with obstinate constipation. In the course of a month, however, she seemed to be in convalescence, but this was of short duration. She again complained of weight and pain in the lower part of the abdomen, accompanied by tenesmus. As lavements were found to go up pretty freely, no suspicion was entertained of any obstruction in the rectum or colon. The symptoms were attributed to internal hæmorrhoids, and leeches were applied to the anus, with oily aperients, and milk diet. But a slow fever continued, with pains in the abdomen, frequent tenesmus, and discharge of considerable quantities of liquid stercoraceous matters. M. Guibert now examined the rectum, and found it blocked up by a mass of hardened fæces, which he extracted, not without much pain to the patient. This was followed by such relief, that hopes of recovery were entertained. But an abscess pointed in the region of the right iliac fossa, which was opened, and a quantity of matter discharged. Thirty-six hours afterwards the patient died. On dissection, a vast abscess was discovered, beneath the peritoneum, and extending from the kidney to the crural arch of that side. M. Guibert thinks that this suppuration was occasioned by the pressure of hardened fæces in the sigmoid flexure of the colon, and in the rectum.

---

59. Pathological Observations.

Dr. Horner, who must be favourably known to our readers, has introduced a few remarks on certain points of pathology, in a recent number of our very esteemed transatlantic cotemporary, (American Journal of the Medical Sciences,) from which we shall make one or two short extracts in the present article.

Dr. H. observes that, on pushing a fine injection into the arteries, he has repeatedly filled the whole venous system, so as to display all the fine meshes under the skin, and to infiltrate the body completely. Judging from these experiments, he is disposed to think "that some of the phenomena of inflammation arise mechanically, and that the substance effused from vessels is, in a measure, according to the mass and momentum of blood flowing through them."

"Thus when irritation determines an increased afflux of blood to a part, if the calibres of vessels are not large enough to permit it to pass freely from the arteries into the veins, serous infiltration first of all occurs: if the afflux be augmented, then coagulating lymph, the particles of which are larger, is effused; and if there be a further augmentation of afflux, the red particles of blood are then effused through the lateral porosities of the vessels. The corresponding phenomena in fine injections, are, first the water, then the size, and lastly the colouring matter, from its particles being the coarsest of the mixture.

"Though many dropsical effusions may be traced to irritation, yet I am disposed to think that some very great errors have been incorporated with their pathology, from the desire to adapt all the phenomena to one standard, to wit, inflammation. This at least I know, that in fine injections of whole adult dropsical subjects, no resistance scarcely is offered by the blood-vessels, and that the injected fluid escapes from them by their lateral parietes or porosities, as fast as it can be thrown in; manifesting thereby evidently a great laxity in their texture. This escape is generally in the order in which we see dropsies to occur, first in the ankles and feet, then up the lower extremities to the trunk; in the hands and wrist, and then up the pectoral extremities to the thorax."

It will be in the recollection of many gentlemen, and indeed in that of our readers, that a physician of this metropolis, maintained, in the Medico-Chirurgical Society, that ABSCESS OF THE LUNGS was a common occurrence. Let us hear what Dr. Horner, who has the most ample means of prosecuting *post mortem* researches, in the Philadelphia Almshouse, says on this subject.

"It is owing to the blood-vessels of the lungs being so superficial, that, as in the intestines, their inflammations pass off either by an increase of their natural secretion of mucus, or by the effusion of serum and of blood. LAENNEC has said, (vol. i. p. 116,) that a collection of pus in the pulmonary tissue in consequence of inflammation, is one of the rarest of cases, at least it is one hundred times more rare than a vomica from tuberculous matter, and a thousand times more so than empyema. In all the dissections of lungs that I have made, *I have met with it but once*, and that lately, (June 29th, 1827,) at the Alms-house, in which case the surrounding part of the lung was gangrenous."

We leave the physician above-mentioned to ponder on these testimonies; and we are inclined to think that he will be more cautious, in future, how he opposes, by an ipse dixit, the laborious investigations of those who are in pursuit of science and truth.

### 60. LONG RESIDENCE OF A BULLET IN THE SKULL.

Baron Larrey lately presented to the Royal Academy of Surgery the cranium of a soldier, who died of phthisis a few weeks previously. This man received a musket bullet in the forehead, a little above the left eye, in the battle of Waterloo. He fell senseless on the ground, and there he remained, without any assistance, for the space of 48 hours. He was then discovered to be alive, and carried to the hospital at Brussels. Various efforts were made to extract the ball, but without success. It appeared to be lodged in the bone, half within and half without the cranium. There were evident symptoms of compression, including paralysis of the right side of the body. Bleeding and the antiphlogistic regimen were rigidly enforced; in process of time the symptoms were mitigated, and he so far recovered as to be sent to Paris. There he got so well as to resume his military duties, and died at last of phthisis, the ball still lodged in the cranium. The only phenomenon that remained, as a consequence of the wound, was the loss of memory in respect to proper names, and the names of nouns substantive. The ball is still seen lodged in the bone, partly within and partly without the cranium. The inner table of the skull had evidently been fractured into several pieces.

### 61. STETHOSCOPE.

Under this head, "one of the New School," has attempted, in the 14th number of the Gazette, to turn into ridicule the investigation of diseases by means of auscultation. The anti-auscultators (and he as the principal) have been beaten out of the field by fair argument—and now, as a "dernier resort," they have recourse to *anonymous* ridicule! The game is up with the anti-auscultators; and we venture to predict, that not a single individual, in this country, will ever attempt to impugn the measure, affixing his real name to the document. Much do we regret, that a cotemporary, whom we esteem, should have given insertion to an anonymous paper tending to retard the advance of a science which needs every auxiliary to rescue it from the reproach of empiricism! To arguments, or even satire, with a responsible signature, every journalist has a right to give currency; but we question the right, or, at all events, the propriety, of putting *anonymous* dealers in ridicule on so respectable a footing.

62. Purpura Hæmorrhagica treated by Venesection.

A case of this kind has recently been reported from Bartholomew's Hospital, which shews the inflammatory nature of this mysterious disease—at least in some instances.

A man, 37 years of age, was admitted under Dr. Latham, having every part of the body sprinkled over with purpurous spots—the gums livid, spongy, and oozing blood—the whole tongue livid, and half of it presenting the appearance of a "large, black, bleeding fungus" shooting from its surface, the inner surface of the cheeks presenting similar phenomena.* The countenance was sallow—the eyes tinged with bile—blood, and nothing but blood, passing by stool. Yet the patient felt strong—had an appetite greater than natural—the urine was free from blood, and the body exhaled a fetid odour. Under these circumstances Dr. Latham had him bled to fifteen ounces—the blood exhibiting a prodigious buffy coat. He was kept on water gruel—had a few doses of aperient medicine containing calomel or the hydrargyrus cum creta, under which treatment, the purpura and hæmorrhage gradually subsided, and the patient recovered. During convalescence he required active purgation, and the use of brisk purgatives to check the inflammatory diathesis and obviate constipation of the bowels.

63. Dandy Fever.

A most extraordinary epidemic, or influenza, under the above ludicrous name, has lately pervaded almost the whole of the West Indian Islands. Whenever it entered a house, no individual in that house escaped. The peculiarity of this epidemic consisted in the fever being always accompanied or succeeded by most severe rheumatic pains in various parts of the body, which threw the patients into such *contortions*, that the name of dandy fever was applied to it. We believe that few deaths were occasioned by the epidemic, but an immense amount of suffering has been entailed on those who caught the influenza. We this day saw an officer who became affected with the fever two days after sailing from Jamaica. The fever lasted only two or three days, but the rheumatic pains, in various parts of his body and extremities, have continued to harrass him day and night, and are rather on the increase since he landed in England. The sheaths of the tendons of his hands have evidently experienced rheumatic inflammation, and effusions have taken place resembling irregularly shaped ganglions. We shall be anxious to have some details of this remarkable epidemic. That it was produced by some atmospheric impregnation there can be little or no doubt—and if so, it strongly supports the doctrine now advocated by Dr. Macculloch, that rheumatic as well as other fevers (not contagious) are the products of malaria.

64. Trephining—Puncture of the Dura Mater.

In a long and very important paper, on Injuries of the Head, lately read at the Medico-Chirurgical Society from the pen of Mr. Brodie, the following, among many other interesting cases, was related.

A woman fell down into a cellar in Monmouth-street, and pitched on her head. She lay senseless, and apparently in profound coma or apoplexy. Mr. Ogle, of Great Russel-street, was summoned, and examined the head, after it was shaved, with great care, but could discover no mark of fracture or other injury. When pressure, however, was made on a particular portion of one of the parietal bones, the patient appeared to evince some signs of sensibility. After employing the usual means, and waiting a rea-

* We have several times had opportunities of ascertaining that these appearances are produced by the coagulable lymph of the blood that has oozed from the vessels and formed itself into these crusts, which tenaciously adhere to the parts. They may be removed by a blunt knife, when the surface beneath will be found unbroken. These phenomena always indicate an inflammatory diathesis and sizy blood. Whenever they present themselves, venesection may be safely employed.—*Ed.*

sonable time, without symptoms of returning sensibility, Mr. Ogle determined to apply the trephine over the part abovementioned. A portion of bone being removed, the dura mater, of a blue colour, bulged out, and was soon on a level with the external surface of the surrounding bone. The sense of touch convinced Mr. Ogle that there was blood effused under the dura mater. He, therefore, punctured this membrane, and blood immediately spouted forth. The patient almost immediately recovered sensibility, and eagerly demanded "what they were about." She recovered.

Mr. Brodie considers it a dangerous practice to puncture the dura mater; but he thinks that such extreme and dangerous cases as that just detailed, warrant the occasional adoption of such a measure.

---

### 65. MIDWIFERY. VERBUM SAT.

Dr. King, of Glasgow, has recently published, through the medium of our northern cotemporary, some cases in midwifery, which are by no means devoid of interest. We are unable, however, to notice these cases at present. The following extract will shew Dr. King's sentiments respecting the projected "return to nature," as we have heard it called—namely, the re-employment of women in midwifery, instead of men. After alluding to the insinuation, or rather the assertion, that great immorality is concealed and committed in the practice of man-midwifery, Dr. King remarks :—

"But the good intentions of our author do not end with this prohibitory caution. Having told the ladies of all the bad consequences to be dreaded from any intercourse with men-midwives, and yet being more than half convinced that children must be assisted into the world somehow or other, he proposes to remove all danger to their morals by educating a generation of young women, in whose hands their characters at least shall be perfectly safe. But there should be consistency in every thing. Does he not shudder at the risk to which he will expose his future midwives? Poor innocent women! little can they know, till too late, the misery for which he destines them; for before they can practise, it is plain that they must be educated, and to acquire the necessary knowledge—under whose care does he intend to place them? Why under the care of the very libertines, whose touch, according to his own showing, is contamination. Unhappy creatures! long before they are fit to serve the public, they must practise on one another, or take refuge in the Magdalene. Reformers have abounded in every age and country; and Horace probably alluded to some of the fraternity, when he said—

'Si quis nunc quærat; Quo res hæc pertinet? illuc;
Dum vitant stulti vitia, in contraria currunt.'

The following language is much too strong, as applied to the indiscreet—we might almost say stultiform, letter of a modern advocate for female accoucheurs. But it is so peculiarly applicable *in another quarter*, that we believe there is not a single member of the profession in this country, however astute his comprehension, who will not instantly recognize the *applicability*.

"But calumny of this kind can only be answered in one way. We must 'live it down,' and the morality of the profession must be read in the private history of its members. *Society is not hoodwinked.* If Tartuffe appear, he will *sooner or later be detected*, and his consequent disgrace and ruin will be so complete, that he must be a reckless rogue and a bold one, that would run every risk against such fearful odds. 'Le jeu ne vaut pas la chandelle.'"

---

### 66. PROTRACTED SLEEP FROM CONCUSSION.

A gentleman (we believe it was Mr. Wigan) lately stated two remarkable instances of this kind, in the Medico-Chirurgical Society. The first case was that of a boy, who, while walking along the street, struck his head against a long pole, which another person was carrying. He was stunned, and carried home insensible. Two or three medical men were summoned, and a violent dispute arose about the proper treatment that was to be pursued—some recommending venesection, others stimulants. The father overheard the altercations, and soon settled the question by turning the doctors to the right about, and determining to leave the case to Nature.

The boy lay as in a profound sleep for seventeen days, during which he was nourished by injections of broth, and some beef-tea got down his throat with difficulty. At the expiration of this period, the boy awoke, unconscious of what had passed, and without a single morbid symptom.

A nearly similar case happening to occur soon afterwards, the gentleman was induced, from recollection of the former instance, to recommend the Hippocratic practice. In this case, the patient continued *three weeks* in a state of profound sleep, and then recovered, without any bad symptom.

---

67. INJURY RECEIVED IN DISSECTION, WITHOUT THE OPERATOR BEING WOUNDED. By SIR ASTLEY COOPER.

The terrible consequences that have resulted from wounds received on dissection are too well known to require notice here, but it is not generally known that distressing effects are sometimes produced without any wound or abrasion upon the hands of the operator. Sir Astley Cooper says " It would seem that under certain circumstances a poison is produced sufficiently strong to excite inflammation, even when there is no wound," and he relates the following case :—

" Mr. Cook, surgeon, at Marsh-gate, Westminster Bridge, sent to me whilst labouring under the highest irritative fever, in consequence of having opened the body of a person who had died of puerperal fever. When I examined him, I found the extremities of his fingers of both hands inflamed, as if they had been dipped in scalding water, and the absorbents of his arms red, hard, and knotted to the axilla ; yet he had not any wound or abrasion of any kind upon his hands, and it would therefore seem that the fluid produced in the abdomen of this woman, in which his fingers had been frequently immersed was of a highly stimulating nature."—*Lectures on Surgery, Vol.* III.

M. Bégin relates a somewhat similar case. We think this case offers incontestible proof that there is a poison generated in the dead body, and that all does not depend on mal-habit of the dissecter, as has been maintained.—*Ed.*

68. ROYAL EXCHANGE OF IDEAS.

In the 2nd CONVERSAZIONE at the ROYAL COLLEGE of PHYSICIANS, Dr. Macmichael was kind enough to entertain and instruct the numerous assemblage by a paper on vaccination, in which a rapid outline of the history, not only of this, but of inoculation, was introduced. The paper was not badly calculated to produce the EFFECT which was desirable on such an occasion. Lady Montague's letters still afforded some amusement, especially those passages in which she doubted the practicability of introducing inoculation in England, for want of a philanthropic physician, who might prefer the good of society to his own private emolument. This was the ARGUMENTUM AD HOMINEM as every one in the assembly felt conscious how much her Ladyship had mistaken the medical character. Instead of experiencing any difficulty in finding a *disinterested* physician in the present day, it would be extremely difficult to point out a single instance where the public good is not unequivocally preferred to self-interest! The consciousness of this *fact*, we say, put the whole company in the best possible humour ; and this pleasant state of feeling was not diminished by the annunciation of another *fact* which beautifully illustrated the philosophical doctrine, that " out of evil cometh good"—or the doctrine of Bernardine de Saint Pierre, that —" all contrasts produce harmonies." We shall not stop to illustrate these delightful views of human nature and human affairs, but proceed at once to the case in point. Dr. M. read an extract of a letter from some " Wise Men in the East," who were led, on failure of vaccine matter, to institute certain experiments, " by which it has been *discovered* that, by inoculating a cow with small-pox matter from the human body, *fine active vaccine virus* is produced." We agree with our esteemed cotemporary, the MEDICAL GAZETTE, that, *if* this should be true—" there can no longer be any fear that we may, at any time, be deprived of the means of combating the small-pox, *since that baneful contagion will furnish its own antidote.*" It is with extreme reluctance that we venture to disturb the train of pleasing anticipations which must naturally flow from this discovery. But we are compelled to remind Dr. Macmichael that this same experiment has been repeatedly tried in this country—for instance, at the Veterinary College, and without the slightest indication of success.

We again congratulate the profession on the establishment of these Conversazioni, which bid fair to re-establish harmony in medical society. Had we an Addison or a Steele in our degenerate times, these immense congregations of the opiferi, salutiferi, and lethiferi of the day, would furnish many an amusing lucubration for—spectators and tatlers! Where are those modern alchymists, the *nimble-fingered* tribe, who can *steal* the words out of our mouths—

> "Calidum, quicquid placuit, *jocoso*
> condere *furto*"—

who can transmute, with the rapidity of lightning, imponderable thought into massive gold? Here is a field for their industry, the like of which was never before laid open to cultivation. Not a subject in the wide domain of medical science and literature that is not here discussed, from the sublime investigations of phrenology, down to the minutest researches in therapeutics—from bumps on the head, to bunnions on the toes—from the *composition* of the mind, to the composition of a corn-plaster—from physic to physics—and from physics to metaphysics—all branches and parts have their respective representatives and professors in these peripatetic or intellectual bazaars. Is Wakley gone to sleep under the *shadow* of his fig-tree? Does he think that Dr. Blundell's lectures will never have an end—or, if endless in themselves, will they not be the end of his readers? Can Mister Wakley not get some good-natured Fellow to *enter* a reporter at the College, under the disguise of a Licentiate? The price of the licence will be repaid fifty-fold before the season is over.

Should the president frown, or the censors threaten, it will be all in vain. They will find themselves in the same predicament, as did Mr. Abernethy, when the Modern Mercury stole his lectures—or, as did Apollo, when the Elder Mercury stole his bullocks!

> Te boves olim nisi redidisses
> *Per dolum* amotas, puerum menaci
> Voce dum terret, *viduus pharetra*
> Risit Apollo.

By the way, we clearly recognised the Gold-headed Cane in the midst of the crowd, artfully *disguised*—having changed its profusion of *golden* tresses for sparse and *silvery* locks. The next edition of its Memoirs will, no doubt, be greatly enriched by the anecdotes which it will be enabled to pick up in the peripatetic Bazaar.

A curious and somewhat alarming phenomenon occurred soon after ten o'clock in the last Conversazione at the College. In the midst of the intellectual exchange—when transfers were extremely brisk—and when (to use the language of another Royal Exchange) there was great "elasticity" in the market, the lights were observed suddenly to "burn blue," and, in a very few minutes, they were so nearly extinguished, that the company could scarcely recognize each other! A portion of the visiters made a scramble for their hats, and decamped with great rapidity; but the more philosophic party imitated Pliny, and determined to see the end of this strange phenomenon. All wits, as well as "all the talents," were instantly at work on the etiology of this sudden obfuscation. Was it a "broad hint" to make themselves scarce? This could not be; for—"their hour was not yet come." Was it typical or emblematical of the *times* we live in, and of the mental adumbration which has spread over the medical horizon during the last three or four years? No. Was it some licentious, but discontented wag, who had bribed the turn-cock, and was playing off a trick on the College? This theory was held to be probable—but a more general opinion prevailed, that, the Independents were at work—and that either Dr. Harrison, or "a blast from the North," was cutting off the supply of hydrogen gas from the College. Just as darkness was becoming visible, and a general *gloom* was pervading every countenance, a flood of ignition burst from every tube of the magnificent chandeliers—and all again was harmony and enlightened conversation! The phenomenon has not yet been accounted for; but a writ of trover will, no doubt, be sued out, in order to discover the author of this *dark* conspiracy.
